# Consensual improvement actions for the Tuberculosis Control Programme in Pernambuco state, Brazil: an e-Delphi study

**DOI:** 10.3934/publichealth.2019.3.229

**Published:** 2019-07-12

**Authors:** Simone Santos Bezerra, Mara Pereira Guerreiro, José Lamartine Soares Sobrinho

**Affiliations:** 1 Center of Biological Sciences, Post-graduation Programme in Therapeutic Innovation, Federal University of Pernambuco, Cidade Universitária, Recife, Pernambuco, Brazil; 2 Unidade de Investigação e Desenvolvimento em Enfermagem (ui&de), Escola Superior de Enfermagem de Lisboa, Parque das Nações, Av. D. João II, 1990-096, Lisbon, Portugal; 3 Centro de Investigação Interdisciplinar Egas Moniz (CiiEM), Instituto Universitário Egas Moniz,Quinta da Granja, 2829, 511 Monte de Caparica, Almada, Portugal; 4 Department of Pharmacy Health Sciences Center, Federal University of Pernambuco-Av. MoraesRego, 123, Cidade Universitária, Recife, Pernambuco, Brazil

**Keywords:** Tuberculosis, health programs and plans, quality improvement, Delphi technique

## Abstract

Objectives: Tuberculosis (TB) remains a major public health problem, particularly in low and middle-income countries. The aim of this study is to consensualise improvement actions for the Tuberculosis Control Programme of the Pernambuco state (SPTC), Brazil. Methods: Firstly, a preliminary workshop was conducted with experts (n = 8), including key stakeholders and health professionals, to select structure and process indicators pertaining to the tuberculosis control programme. Then, an e-Delphi was carried out with a purposive sample of 11 local TB experts. The first-round questionnaire was comprised of 19 open-ended questions on possible improvement actions, based on programme indicators obtained in the previous stage. In the second-round experts rated each action for relevance and feasibility, using a four-point scale. In the last round the participants rated the actions again, in the light of group's answers. We used published criteria to define consensus at the outset of the study. Key findings: Eighty-nine improvement actions achieved a high degree of consensus in both feasibility and relevance in round three. Eighty-six actions were grouped under 19 structure and process indicators, while three were consideredcross-sectional in scope (i.e. related to more than one indicator). Ten out of the 86 actions obtained at least 70% of ratings on the highest score of the scale both for relevance and feasibility. These included: “Request and availability of sputum pots can be made by any health professional in the health unit”. Conclusions: The wide array of actions obtained in this Delphi represent a resource from which local SPTC services can select the actions most suitable for each context. The ten most relevant and feasible actions represent a particularly useful starting point to streamline change and potentially improve programme indicators.

## 1. Introduction

Tuberculosis (TB) remains a major health problem, deserving considerable attention, particularly in low and middle-income countries [1],[2].

In Brazil, disease control actions are part of the National Tuberculosis Control Programme (NTCP), which is developed in the public network of the health system (Sistema Único de Saúde -SUS). This programme provides universal access to TB prevention, surveillance and management. In 2001, the programme became decentralized and the responsibility of NTCP actions was transferred to primary health care. At a municipal level, the NTCP is operationalized together with the Family Health Programme Strategy (FHS) [3]. FHS provides primary care for defined populations throughmultidisciplinary healthcare teams, comprised by a physician, a nurse, a nurse assistant and four to six full-time paid community health workers, who are lay members. Each team is organized geographically, covering up to 1000 households [4]. Depending on its nature, NTCP actions can be implemented in different sites: in people's homes, by FSH members (e.g. giving information about the disease), in Basic Health Units (e.g. referring patients for laboratory tests), or in outpatient units by referral from primary care (e.g. treatment).

A global fall in TB incidence and mortality rates has been achieved. Among the 22 countries accounting for 80% of global cases, seven have reached the reduction targets of incidence, prevalence and mortality for 2015, including Brazil. Nonetheless, challenges remain, such as treatment discontinuation and a greater TB incidence among high-risk populations [5],[6]. In the State of Pernambuco and its capital city, Recife, the rates for TB-related mortality, incidence and treatment discontinuation have not yet reached the targets set by the Ministry of Health [7]. Therefore, efforts need to be directed to improve patient care, disease control and, consequently, reduce TB burden.

## 2. Materials and method

We chose the Delphi technique, a formal consensus technique that takes place in a series of sequential mailed questionnaires to isolated experts interspersed by controlled feedback [8]. The literature provides a plethora of Delphi forms and offers examples of its use in tuberculosis research [9],[10]. One Delphi form is the policy Delphi, in which experts agree future policy on a given topic [9]. In our study we used TB control indicators, obtained in a preliminary workshop consultation, as a starting point to derive improvement actions. The Delphi was conducted between October 2015 and September 2016.

### 2.1. Preliminary work

The indicators were pre-selected in a workshop with eight participants (two pharmacists, one nurse plus two physicians with experience in TB management, a nurse from a local epidemiological centre and the coordinator of the local tuberculosis control programme).

The starting point was a set of 27 indicators, which included the TB control indicators recommended by the Brazilian Health Ministry, as well as indicators for evaluating TB programmes proposed by Scatena and co-workers [11]. Participants were asked to rate the relevance of each indicator for evaluating the SPTC using a three-point scale (relevant, irrelevant or unsure). Consensus was used to accept and reject indicators; consensus was defined as at least 75% of the participants rating indicators as relevant or irrelevant, respectively. Failure of an indicator to meet these criteria meant consensus was not achieved. Participants were also asked to suggest new indicators to evaluate the local tuberculosis control programme.

Twelve of the 27 indicators failed to achieve consensus as relevant for evaluating the tuberculosis control programme in Pernambuco. The remainder, presented in Table 1, were consensus-approved. Table 2 presents the four indicators suggested by this panel.

**Table 1.** Indicators approved in the preliminary phase.

Number	Description
1	Proportion of patients depending on motorized transport for access to medical consultation and TB medication.
2	Number of days with lack of medication during treatment.
3	Proportion of patients whose medical care is performed in the health service closer to home.
4	Proportion of patients who have access to medical consultation in less than 24 hours for emergency situations.
5	Proportion of patients who are visited by health professionals at home.
6	Proportion of patients receiving information about tuberculosis (TB) and its treatment.
7	Proportion of communicants or contacts (people living with the patient) who receive information about TB and its treatment.
8	Proportion of patients who participate in TB groups in the Health Service.
9	Number of advertisements/campaigns/educational work performed semiannually by professionals of the Health Service.
10	Number of community actions carried out every six months for delivery of the sputum pot.
11	Percentage of culture tests performed among total retreatment cases.
12	Percentage of new smear positive pulmonary cases in directly observed treatment (DOT).
13	Percentage of contacts of patients with pulmonary tuberculosis who are examined for the disease.
14	Proportion of HIV tests conducted among new cases of tuberculosis.
15	Percentage of retreatment of the total cases.

**Table 2.** Indicators proposed in the preliminary work.

Number	Description
16	Proportion of treatment discontinuation in new cases of pulmonary TB (%).
17	Proportion of patients who have access to TB medication in a timely manner.
18	Proportion of patients who do not adhere to treatment.
19	Proportion of under-reported tuberculosis cases.

### 2.2. Delphi panel

#### 2.2.1. Sampling

Panel members were recruited from the CNPQ Lattes Platform, a database maintained by the Brazilian government with curricular information of researchers working in Brazil and abroad. The search was performed by subject, using the term “tuberculosis”. We employed purposive sampling to ensure the selection of an adequate range of experts, with an understanding about the area and a variety of viewpoints [12]. An expert was defined as a health professional from the public or private sector, practicing in Pernambuco, with knowledge and/or practical experience in TB management. These criteria were operationalized by selecting subcategories of health professionals in the Lattes Platform, followed by curricula evaluation.

Experts were invited to take part in the study by e-mail, with the aid of an information leaflet. Those wishing to participate were ask to fill in and return a participation form.

#### 2.2.2. Data collection

We defined at the outset the number of rounds (three), based on the study objective, resources available and estimated sample burden. Electronic questionnaires were prepared for all rounds, employing Google forms.

The first-round questionnaire was comprised of 19 open-ended questions; each question pertained to a structure or process TB control indicator obtained in the previous phase. Participants were asked to suggest actions to improve indicators results or to achieve the national Health Ministry official target (%), where applicable. The questionnaire was piloted in a convenience sample of two pharmacists and two nurses.

The second-round questionnaire contained the actions suggested by the panel in the previous round, listed under the respective indicator. Moreover, for one indicator (“Proportion of treatment discontinuation in new cases of pulmonary TB”), we added an action with effectiveness underpinned by robust evidence [13]: “Establish ‘pre-appointment’ reminders (visits, phone calls, letters or SMS before scheduled appointment)”. Participants were asked to rate each action for feasibility and relevance based on a four-point scale used in a policy Delphi [14], described in Tables 3 and 4. The questionnaire had also space for comments.

**Table 3.** Feasibility scale.

Rating	Label	Description
3	Definitely feasible	No interference or impediment to implementation; independent of any condition for its development; acceptable for all stakeholders.
2	Feasible	Few interferences or impediments to implementation; depends on few conditions for its development; some effort required to convince stakeholders.
1	Unfeasible	Many interferences or impediments to implementation; depends on many conditions for its development; hardly acceptable for stakeholders.
0	Definitely not feasible	Appears impossible to implement; depends on several issues for development; unacceptable for stakeholders.

**Table 4.** Relevance scale.

Rating	Label	Description
3	Very relevant	It will have very positive effects and no negative effect; extremely beneficial; justifiable on its own merits. Extremely useful for programme improvement.
2	Relevant	It will have positive effects and few negative effects; beneficial; justifiable as a supplementary measure or together with other actions. It has some utility for programme improvement.
1	Not relevant	Will have a negative effect; harmful; little justifiable. Unlikely to be useful for programme improvement.
0	Irrelevant	It will have a great negative effect; extremely harmful; unjustifiable. Totally useless for programme improvement.

We defined consensus at the outset of the study resorting to a published classification [15], used by others in policy Delphi studies [14]. It was deemed that actions meeting a medium or high degree of consensus in both feasibility and relevance would be accepted or rejected, depending on whether consensus was reached on positive or negative points of the scale (Table 5). Actions with low or no degree of consensus would not be accepted.

**Table 5.** DeLoe's classification for consensus using a 4-point rating scale.

Degree of consensus	Distribution of ratings
High	At least 70% of ratings in one point or 80% in two related points^(*)^
Medium	At least 60% of ratings in one point or 70% in two related points^(*)^
Low	At least 50% of ratings in one point or 80% in two related points^(*)^
None	< 50% of ratings in one point or < 60% in two related points. ^(*)^

Note: (*) Related points are those positive (points 3 and 2) or negative (points 1 or 0).

In the third round we asked experts to re-rate actions for relevance and feasibility in light of the group's answers. Due to the extensive nature of the questionnaire we restricted the number of actions included, by selecting only those that did not reach a high or medium degree of consensus in the second round. Personalized questionnaires were e-mailed, comprising the ratings of each panelist and the group's median rate for each action. Space for comments was also provided.

#### 2.2.3. Data analysis

Textual data emerging from the first round was analysed by two of the authors. It involved systematically reading the suggested actions, discussing similarities and differences in content, merging actions with similar content and harmonising wording across all actions. Numerical data collected in the second and third rounds were subjected to descriptive statistics with the aid of SPSS release 11.5.

**Figure 1.**
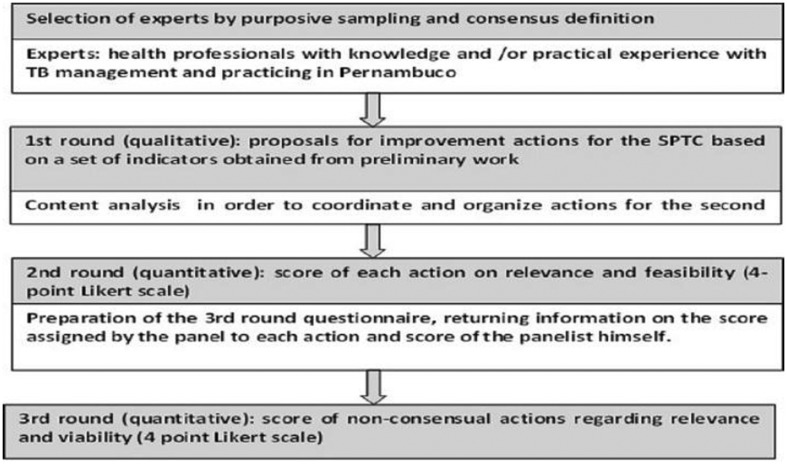
Provides an overview of the e-Delphi.

## 3. Results

Analysis of 395 curricula in Lattes platform led to the selection of 70 experts. Nineteen agreed to participate in the study (27.1% response rate) but only 10 answered the first round. Eleven experts answered the second and the third-round questionnaire, comprising therefore the study sample. No attrition occurred between the second and the third round.

### 3.1. Panel profile

The panel was comprised by nurses (54.5%, n = 06) and pharmacists (45.5%, n = 05). Most panelists were female (90.1%, n = 10), aged between 31 and 56 years old (median = 35.5 and standard deviation = 7.4). The minimum academic degree was of a specialisation in public health and epidemiology (63.6%, n = 7). Ten out of 11 panelists had five years or more of experience with tuberculosis patients or with the programme, SPTC (median = 6 years; standard deviation = 3). All panelists worked in the public sector.

### 3.2. First round

Analysis of panel contributions yielded a total of 93 actions distributed under 19 indicators.

### 3.3. Second round

Eighty-three actions obtained a high degree of consensus for relevance and feasibility (i.e. 70% of ratings in one positive point of the scale or 80% in the two positive points).

The ten actions with a medium or low degree of consensus were subjected to the next round. No additional comments were received pertaining to the actions.

### 3.4. Third round

Consensus building led to the approval of six out of the ten actions which previously obtained medium or low degree of consensus (Table 6). This represents a total of 89 actions approved by consensus; three were deemed cross-sectional in scope whilst the remainders were grouped under 19 structure and process indicators. The full list of improvements actions is available from the authors upon request.

**Table 6.** Actions not approved by consensus at the end of the Delphi panel.

Indicator	Action
Indicator1: Proportion of patients depending on motorized transport for access to medical consultation and TB medication.	Action 3: Delivery medication at patients' homes through community health workers.
Indicator 5: Proportion of patients who are visited by health professionals at home.	Action3: Reduce the number of families per family health team.
Indicator 12: Percentage of new smear-positive pulmonary cases in directly observed treatment (DOT).	Action 2: Increase the number of professionals participating in the directly observed treatment (DOT), and provide financial incentives to community health workers for each documented DOT.
Indicator 16: Proportion of treatment discontinuation in new cases of pulmonary TB (%).	Action 6: Establish “pre-appointment” reminders (visits, phone calls, letters or SMS messages before scheduled appointment), by resorting to health services' staff, voluntary or community members.

Finally, ten of the 89 actions reached at least 70% of ratings on point three of the scoring scale both for relevance and feasibility (Table 7).

**Table 7.** Improvement actions with at least 70% of ratings on the highest positive score of the scale both for relevance and feasibility (third round).

Indicator	Action
Indicator3: Proportion of patients whose medical care is performed in the health service closer to home.	Action 2: Promote initial training and continuing education for professionals in Basic Health Units / Family Health Units on diagnosis, treatment and monitoring of TB.
Indicator 6: Proportion of patients receiving information about TB and its treatment.	Actions 1: Conduct training of health professionals to offer information about TB and its treatment coupled the active search of symptomatic respiratory patients and home visits. 2. Implement group education about TB and its treatment by multidisciplinary teams, for community members in general 6. Provide individual education to patients and also the consultation with a health professional, promoting listening and bond.
Indicator 7: Proportion of communicants or contacts (people living with the patient) who receive information about TB and its treatment.	Action 2: Contact people living with a person with TB once a positive diagnosis is established, to perform diagnostic tests and/or chemoprophylaxis (depending on the situation).
Indicator 10: Number of community actions carried out every six months for delivery of the sputum pot.	Actions 2: Search patients with respiratory symptoms during home visits and make sputum pots available to symptoms-positive patients. 6. Train all health professionals of basic health units who take part in community actions in identifying people with respiratory symptoms and their contacts.
Indicator 11: Percentage of culture tests performed among total retreatment cases.	Action 2: Inform and raise awareness on the general public about the importance of the TB culture test.
Indicator 13: Percentage of contacts of patients with pulmonary tuberculosis who are examined for the disease.	Action 6: Allow any health professional in the health unit to request and provide sputum pots.
Indicator 19: Proportion of under-reported tuberculosis cases.	Action 2: Raise awareness and train Family Health Strategy teams to report TB cases in a timely manner, in particular through activities carried out by the epidemiological surveillance team, emphasizing the importance of health planning through data obtained from mandatory reporting of infectious diseases problems.

**Figure 2.**
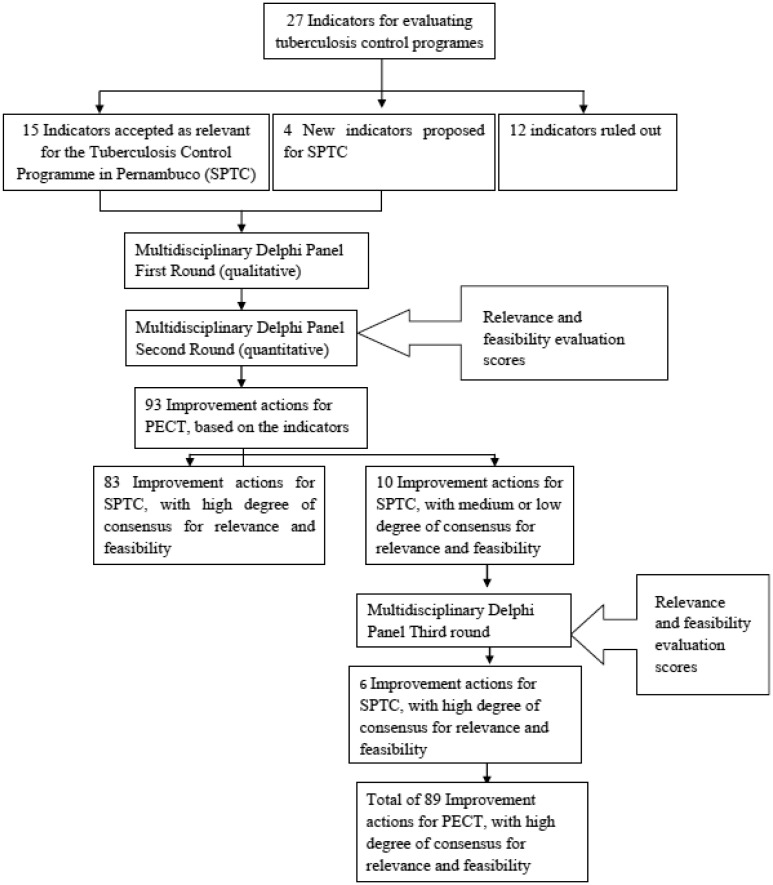
The results of the Delphi panel.

## 4. Discussion

This study was set up to consensualise improvement actions to the SPTC. We obtained 89 consensual actions, ten of which obtained simultaneously the highest score for relevance and feasibility by at least 70% of the panel. This means that experts considered that there is no interference or impediment to their implementation, they do not depend on any condition for their development and they have great acceptance by stakeholders. Experts endorsed that they will have very positive effects and no negative effect, they will be extremely beneficial, they are justified by their own merits and they will be extremely useful for improving the programme.

Only four of the improvement actions failed to reach consensus. One of these action—“Establish ‘pre-appointment’ reminders (visits, phone calls or letters or SMS) before scheduled appointment”—has proven effectiveness in avoiding treatment discontinuation, but our expert panel did not agree with its feasibility in the local context of Pernambuco. A possible explanation for this may be budgetary constraints and insufficient numbers of health professionals.

Consensus was not achieved either for the action “Increase the number of professionals participating in the directly observed treatment (DOT), and provide financial incentives to community health workers for each documented DOT”, which could reduce the percentage of new smear-positive pulmonary cases in patients enrolled in DOT. These results may be explained by the controversy around the effectiveness of DOT in TB treatment. In a recent systematic review of 11 controlled trials (RCTs), cluster RCTs and quasi-RCTs with a total of 5662 TB patients receiving treatment for active TB or latent TB infection, DOT did not improve cure or treatment completion [16]. This contrasts with a previous Cochrane review and the evidence [17], albeit less robust, from a systematic review and meta-analysis of observational studies on strategies for reducing treatment discontinuation in drug-resistant tuberculosis [18]. In the latter, DOT provided throughout the treatment course in 36 out of the 75 studies included, corresponding to a total of 7635 patients, showed a beneficial effect. This included instances where DOT was provided through community health workers rather than nurses or health care professionals in a facility [18]. It has been noted that although DOT is costly, it may have an impact on the early detection of adverse drug reactions, an aspect that has not been considered by Karumbi and Garner's work [16],[19]. Moreover, self-administered treatment may be challenging to patients with lower educational background, as often observed in low-to-middle income countries [19].

Another nonconsensual action was “Reduce the number of families by family health team”, in order to increase the “Proportion of patients who are visited by health professionals in their homes”. Lack of consensus on its feasibility may be due to the low proportion of population coverage by family health teams in Pernambuco, which stands at 52.58% [20]. Another possible explanation for this finding is that performance and number of home visits is influenced not only by the population coverage, but also by the resolutivity of family health teams and internal organisation of services [21].

The last non-consensual action obtained was “Delivery medication at patients' homes through the community health workers”, aiming to reduce the “Proportion of patients depending on motorized transport for access to medical consultation or withdrawal of the drug at the pharmacy”. Treatment discontinuation can occur when TB medication is free of charge, as other access factors represent a cost, such as the need to commute and lost work hours [22]. However, increasing access to medicines without close supervision by health professionals may be counterproductive and result in economic waste, adverse drug reactions and increased resistance to anti-TB drugs [23].

With respect to the generalisability of the study, it should be taken in account that indicators and improvement actions proposed for a local programme may be a useful starting point to different countries but cannot be simply transferred, due to differences in professional culture, health care organisation and clinical practice [24]. This study has some limitations. First, the number of participants in the Delphi panel was relatively small. Whilst additional databases could have been used to identify experts (e.g. health workers of the Tuberculosis Program within the State or Municipal Secretaries), response rates in the absence of incentives are uncertain. The ideal number of experts in Delphi panels is unknown; published studies have panels with a size ranging from less than 15 to hundreds of participants [8].

Secondly, our sample composition may be criticized for the lack of experts with a medical background, who are stakeholders in primary care and closely involved in TB treatment. The lack of response to our invitations from physicians (and the overall low response rate from busy professionals) may reflect the fact that no incentives were used. Fry and coworkers quoted empirical evidence supporting the provision of incentives to achieve adequate response rates in surveys targeting health professionals [25]. Offering monetary incentives to improve recruitment and retention rates is ethically legitimate, insofar that recruitment targets are critical to meet studies objectives and deliver socially useful knowledge [26]. The use of research incentives to health professionals, albeit uncommon in Brazil, deserves consideration in future studies.

Finally, although this research adhered to rigorous principles, namely in the definition and selection of experts, results should not be regarded as definitive: they only reveal situations that could be changed, encouraged or made possible [27]. For example, the lack of consensus of an evidence-based action may reflect local circumstances, such as health care organisation, professional culture and clinical practice, or mirror an inadequate judgment. Therefore, improvement actions obtained in this study require discussion by health managers and health professionals on the financial aspects and other practicalities entailed in implementation. Ideally, implemented actions should be subjected to empirical evaluation.

## 5. Conclusion

The wide array of actions obtained in this Delphi represent a resource from which local policymakers and programme managers can select the ones preferred for each context. The ten most relevant and feasible actionsrepresent a particularly useful starting point to streamline change and potentially improve programme indicators. Such actions include promoting training and continuing education for professionals in Basic Health Units/Family Health Units on diverse subjects related to TB; implementing group and individual education about TB and its treatment by multidisciplinary teams; identifying people with respiratory symptoms and their contacts and performing diagnostic tests and/or chemoprophylaxis; making sputum pots available to symptoms-positive patients and raising awareness and training Family Health Strategy teams to report TB cases in a timely manner. Local public health policies on TB may focus these actions firstly. Involving programme health professionals and Family Health Programme Strategy groups in selecting and prioritizing improvement actions may facilitate implementation.
